# Dickkopf-3 (DKK3) in cardiovascular diseases: from a stress-responsive biomarker to a potential therapeutic target

**DOI:** 10.3389/fphar.2026.1842032

**Published:** 2026-10-08

**Authors:** Xiaoyan Fu, Jianfeng Dai, Xiaoqian Wang, Libin Qiu, Yunkai Hua

**Affiliations:** 1 Structural Cardiology Department, Weifang People’s Hospital (The First Affiliated Hospital of Shandong Second Medical University), Weifang, Shandong, China; 2 Electrocardiographic Room, Weifang People’s Hospital (The First Affiliated Hospital of Shandong Second Medical University), Weifang, Shandong, China

**Keywords:** cardiovascular diseases, dickkopf-3, DKK3, renal-cardiac axis, TGF-β, wnt signaling

## Abstract

Dickkopf-3 (DKK3) is a secreted glycoprotein that functions as a context-dependent modulator of the Wnt/β-catenin and transforming growth factor-beta (TGF-β) signaling pathways. Originally studied in development and cancer, DKK3 has recently emerged as a key stress-responsive factor. It is released by epithelial and other cell types under pathological strain and actively participates in fibrotic, inflammatory, and remodeling processes across various organ systems. In cardiovascular medicine, DKK3 is gaining recognition not only as a promising biomarker for renal and vascular injury—conditions closely intertwined with cardiovascular outcomes—but also as a direct mediator in the pathogenesis of specific cardiovascular conditions such as abdominal aortic aneurysm (AAA) and heart failure. This review synthesizes current knowledge on DKK3, beginning with its fundamental biology and established roles in renal, pulmonary, and dermal fibrosis, which provide crucial mechanistic parallels. We then focus on the evolving evidence of DKK3’s involvement in cardiovascular diseases, critically evaluating its biomarker potential for risk stratification in conditions like diabetic kidney disease and its direct pathophysiological roles in vascular smooth muscle cell phenotype switching, myocardial remodeling, and aneurysm formation. We delve into specific molecular mechanisms, including interactions with the ATF6-TGFβ3-Smad2/3 axis and low-density lipoprotein receptor-related protein 1 (LRP1), as revealed by preclinical models. Finally, we explore the therapeutic implications of targeting the DKK3 axis, discuss the challenges posed by its context-dependent dual roles, and outline future research directions necessary to translate these discoveries into clinical applications for cardiovascular patients.

## Graphical Abstract

**Figure:**
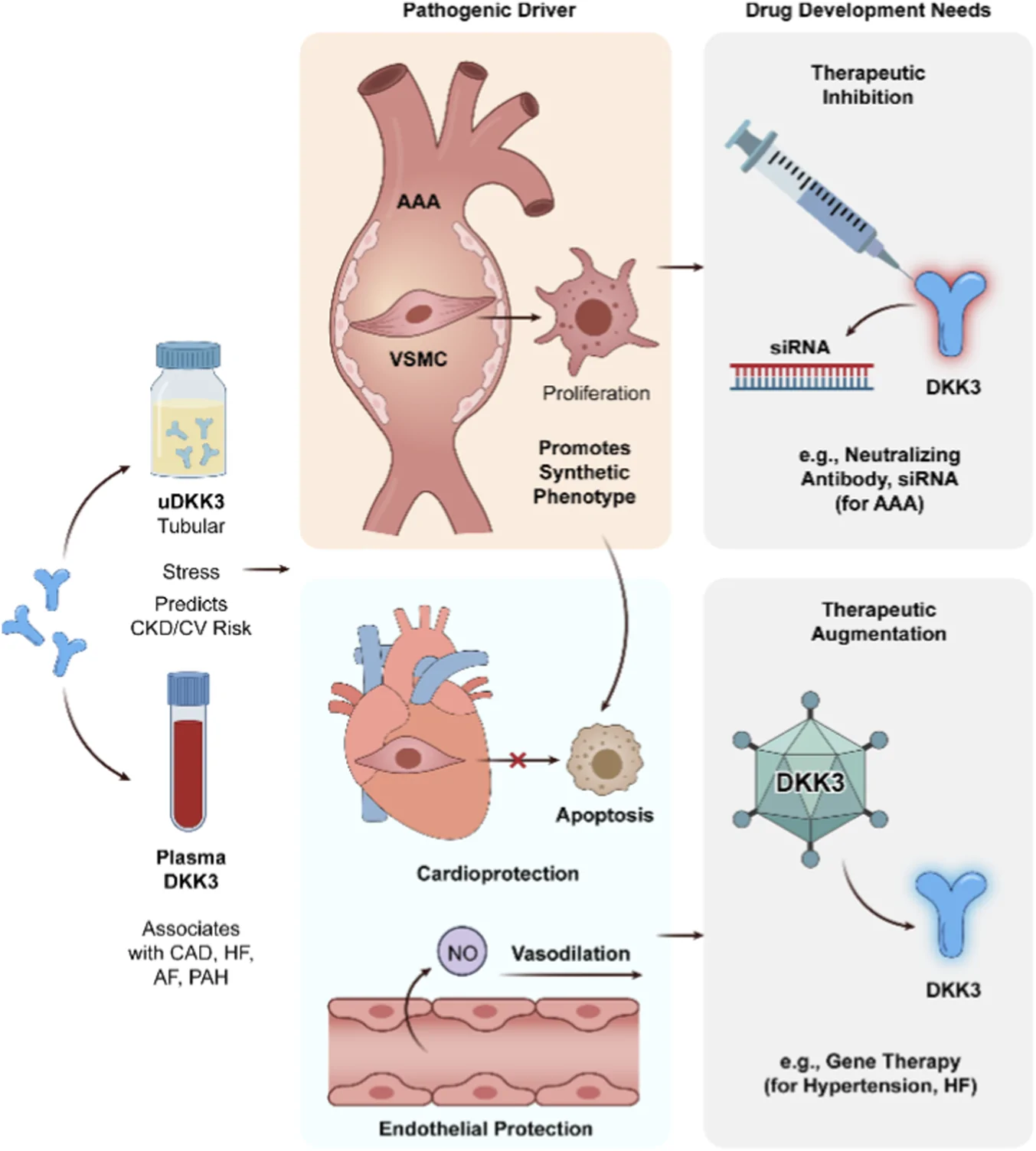
The Multifaceted Roles of DKK3 in Cardiovascular Pathophysiology and Therapeutic Implications. This illustration summarizes the key contexts of Dickkopf-3 (DKK3) involvement in cardiovascular health and disease. Secreted DKK3 acts as a biomarker for organ stress, with urinary DKK3 (uDKK3) predicting renal and cardiovascular risk, and circulating DKK3 levels associating with various CVDs. Its functional role is highly context-dependent. DKK3 is a clear pathogenic driver in abdominal aortic aneurysm (AAA), promoting vascular smooth muscle cell (VSMC) phenotypic switching. Conversely, it exerts protective functions in other settings, such as in the myocardium (inhibiting cell death and fibrosis) and endothelium (promoting repair and vasodilation). This duality informs two complementary therapeutic strategies: Inhibition (e.g., *via* neutralizing antibodies or siRNA) for diseases like AAA where DKK3 is pathogenic, and Augmentation (e.g., *via* gene therapy) for conditions like hypertension or heart failure where DKK3 deficiency or loss-of-function contributes to pathology.

## 1 Introduction

Cardiovascular diseases (CVDs) remain the leading cause of global morbidity and mortality. Their pathogenesis often involves a complex interplay between metabolic dysregulation, chronic inflammation, oxidative stress, and maladaptive tissue remodeling (Global Cardiovascular Risk et al., 2023). Despite advances in pharmacotherapy and interventional cardiology, a persistent need exists for novel biomarkers that can identify high-risk patients at early (Neumann et al., 2024), potentially reversible stages, and for new therapeutic targets that address underlying pathogenic mechanisms like fibrosis and vascular dysfunction. The Dickkopf (DKK) family of secreted glycoproteins, comprising DKK1-4 and Soggy (DKK4), are primarily known as regulators of the evolutionarily conserved Wnt signaling pathway. While DKK1 is the most studied member, acting as a canonical Wnt inhibitor, Dickkopf-3 (DKK3) has garnered increasing attention for its unique, context-dependent functions (Mourtada, Thibaudeau, Wasylyk and Jung, 2023). Unlike other DKKs, DKK3 exhibits a more complex relationship with Wnt signaling, capable of both inhibition and activation depending on the cellular context and receptor milieu. Furthermore, DKK3 expression is markedly induced in parenchymal cells (e.g., renal tubular epithelial cells, keratinocytes, cardiomyocytes) in response to various stress stimuli, including oxidative stress, mechanical stretch, and inflammatory cytokines (Chen et al., 2025). This “stress-response” signature of DKK3 has positioned it as a promising biomarker for subclinical organ damage. Most notably, urinary DKK3 (uDKK3) has been validated as a strong predictor of progressive chronic kidney disease (CKD) and acute kidney injury (AKI), conditions that are tightly linked to adverse cardiovascular outcomes (Roscigno et al., 2021; Schunk et al., 2019). Beyond its biomarker utility, functional studies across multiple organ systems reveal DKK3 as an active player in driving pathological processes, particularly fibrosis, by modulating not only Wnt but also TGF-β signaling, influencing macrophage polarization, and promoting epithelial-to-mesenchymal transition (Kano et al., 2022). In the cardiovascular system, the direct exploration of DKK3 is still in its nascent stage compared to nephrology or oncology (Sun et al., 2025). However, emerging data paint a compelling picture. DKK3 is implicated in the phenotypic switching of vascular smooth muscle cells (VSMCs)—a cornerstone of vascular remodeling in atherosclerosis and aneurysm formation (Karamariti et al., 2018). It is differentially expressed in heart failure models and may influence myocardial fibrosis (Piek et al., 2020). Its role as a ligand for LRP1, a critical receptor in amyloid-β clearance and vascular biology, opens another intriguing avenue related to vascular integrity and disease (Yang et al., 2025).

## 2 Dickkopf-3: an overview of biology and cross-system pathophysiological roles

### 2.1 Molecular biology and signaling of DKK3

Dickkopf-3 (DKK3), also known as reduced expression in immortalized cells (REIC), is a secreted glycoprotein that belongs to the DKK family of Wnt signaling modulators (Mourtada et al., 2023). The human *DKK3* gene is located on chromosome 11p15.3 and encodes a protein that undergoes significant post-translational processing to yield its mature, secreted form. The DKK3 gene spans approximately 80 kilobases and consists of multiple exons. Alternative splicing gives rise to several transcript variants, with the major isoforms being the full-length DKK3 (often referred to as DKK3A) and a shorter variant lacking exon 4 (DKK3B). DKK3A is the predominant and most studied form. The pre-proprotein of DKK3 consists of a signal peptide, a propeptide region, and the mature protein domain. The mature, secreted DKK3 glycoprotein (∼38–40 kDa) is characterized by two conserved, cysteine-rich domains (CRD1 and CRD2), a structural hallmark of the DKK family. However, unlike other DKK members, DKK3 lacks a clear N-terminal signal peptide for classical secretion, instead utilizing an unconventional secretory pathway, a feature that may influence its spatial and temporal bioavailability (Wang et al., 2024). Post-translational modifications, particularly N-linked glycosylation, are critical for its proper folding, stability, and secretion. Interestingly, a C-terminal truncation of DKK3, termed DKK3c, has been identified and appears to have distinct, sometimes opposing, biological activities compared to the full-length protein, adding another layer of complexity to its functional regulation (Medinger et al., 2014). The expression of DKK3 is not ubiquitous; it is developmentally regulated and shows tissue-specific patterns, with high basal expression in the brain, heart, and kidneys. Its expression is highly inducible by a variety of cellular stresses, including endoplasmic reticulum (ER) stress, oxidative stress, hypoxia, and inflammatory cytokines, positioning it as a potent stress-response protein (Chen et al., 2025) (Figure 1). A summary of its constitutive and context-regulated expression across different organs and cell types is provided in Table 1.

**FIGURE 1:**
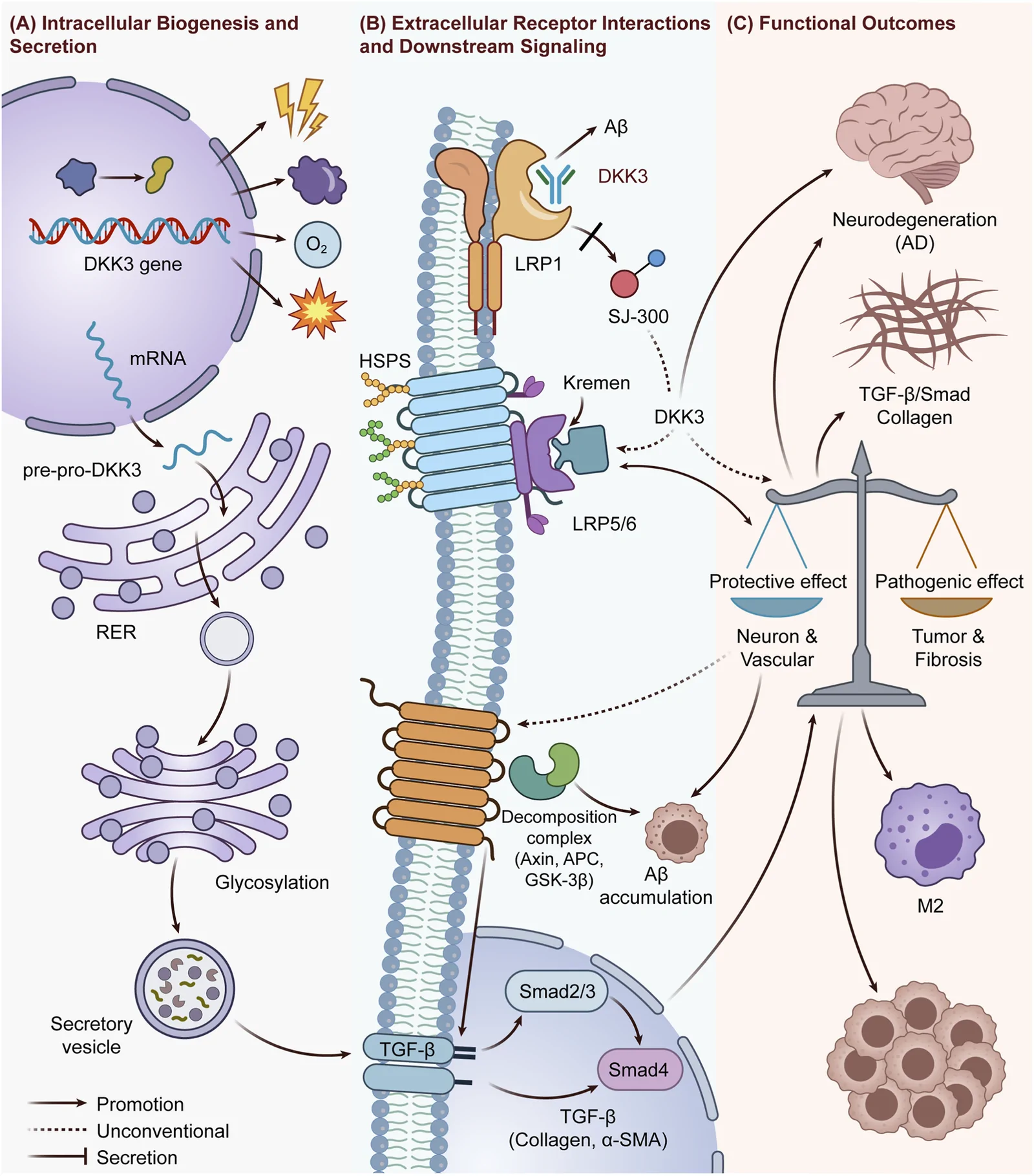
Molecular synthesis, secretion, and multifaceted signaling network of Dickkopf-3 (DKK3). This schematic illustrates the complex biology of DKK3 **(A)** Intracellular Biogenesis and Secretion: Following induction by various cellular stressors (e.g., ER stress, hypoxia, cytokines), the *DKK3*gene is transcribed. The translated pre-pro-DKK3 protein enters the endoplasmic reticulum (ER) for initial folding and N-linked glycosylation, critical for its stability. After further processing in the Golgi apparatus, mature DKK3 is released into the extracellular space *via* an unconventional secretory pathway **(B)** Extracellular Receptor Interactions and Downstream Signaling: Secreted DKK3 engages in context-dependent crosstalk with multiple pathways. (1) Wnt Signaling Modulation: DKK3 can bind the transmembrane protein Kremen, potentially influencing the internalization of the LRP5/6 co-receptor complex, leading to context-dependent inhibition or modulation of canonical Wnt/β-catenin signaling. Its interaction with cell surface heparan sulfate proteoglycans (HSPGs) may also localize or modulate its activity. (2) LRP1 Axis: DKK3 acts as a ligand for the multifunctional receptor LRP1. This interaction can competitively inhibit the LRP1-mediated clearance of ligands like Amyloid-β (Aβ), with implications for neurodegeneration and vascular biology. The small molecule inhibitor SJ-300 selectively blocks this interaction. (3) TGF-β Pathway Crosstalk: DKK3 extensively interacts with the Transforming Growth Factor-beta (TGF-β) pathway, a master regulator of fibrosis. This crosstalk can modulate Smad2/3 phosphorylation and downstream transcriptional responses, often in concert with ER stress sensors like ATF6, driving pro-fibrotic and inflammatory programs **(C)** Functional Outcomes: Through these interconnected axes, DKK3 regulates diverse cellular processes including cell fate determination, survival/apoptosis, migration, and immune cell polarization (e.g., macrophage towards a pro-fibrotic M2 phenotype). Its net effect is highly context-dependent, contributing to its roles in organ fibrosis, cancer biology, and cardiovascular diseases.

**TABLE 1:** Expression patterns of DKK3 in different organs and cell types.

Organ/Tissue	Major cell types expressing DKK3	Expression notes and inducers
Kidney	Tubular epithelial cells, podocytes (lower)	High constitutive expression. Markedly upregulated under stress (e.g., hyperglycemia, ischemia, proteinuria). Secreted form (uDKK3) is a clinical biomarker
Heart	Cardiomyocytes, cardiac fibroblasts, endothelial cells	Moderate constitutive expression. Upregulated in response to pressure overload, myocardial infarction, and metabolic stress. Expression can be protective or pro-fibrotic depending on context
Vasculature	Vascular smooth muscle cells (VSMCs), endothelial cells	Low in quiescent state. Markedly upregulated in synthetic/proliferating VSMCs (e.g., in AAA, atherosclerosis). Induced in endothelial cells by shear stress and inflammation
Brain	Neurons, glial cells	High constitutive expression, particularly in developing brain. Modulated in neurodegenerative diseases (e.g., Alzheimer’s)
Skeletal muscle	Myofibers (as a myokine)	Upregulated in obesity and type 2 diabetes. Secreted DKK3 can impair muscle stem cell differentiation
Skin	Keratinocytes, dermal fibroblasts	Induced by radiation, injury, and inflammatory stimuli. Drives dermal fibrosis *via* paracrine signaling
Liver	Hepatocytes, hepatic stellate cells	Expression changes associated with stellate cell activation and fibrosis
Pancreas	Acinar cells, cancer-associated fibroblasts (CAFs)	Early, transient expression is protective; sustained CAF expression is pro-tumorigenic and pro-fibrotic
Lung	Alveolar epithelial cells, pulmonary vascular cells	Associated with fibrotic and hypertensive pulmonary disease
Immune cells	Macrophages (M2 subset)	Can be expressed by and can polarize macrophages towards a pro-fibrotic phenotype

The DKK family is classically defined by its ability to antagonize canonical Wnt/β-catenin signaling by binding to the Wnt co-receptors low-density lipoprotein receptor-related proteins five and 6 (LRP5/6) (Yang et al., 2025). Here, DKK3 stands apart. While DKK1 and DKK2 bind LRP5/6 with high affinity, DKK3 exhibits only weak or context-dependent binding. This fundamental difference underpins its unique and often paradoxical functional profile. DKK3’s relationship with Wnt signaling is not that of a simple inhibitor but rather of a context-dependent modulator (Ferrari et al., 2019). DKK3 can inhibit canonical Wnt/β-catenin signaling, but frequently through mechanisms distinct from DKK1. In some contexts, DKK3 may sequester the transmembrane protein Kremen, which forms a ternary complex with DKK1 and LRP6 to promote rapid internalization of the receptor (Xu et al., 2020; Zhang et al., 2022). DKK3’s interaction with Kremen, potentially in a DKK1-independent manner, can still influence receptor trafficking and downstream signaling. In cervical cancer, for instance, DKK3 upregulation is associated with β-catenin degradation and tumor suppression, highlighting its role as a Wnt pathway inhibitor in certain malignancies (Lu et al., 2025). Paradoxically, DKK3 has also been reported to activate Wnt signaling. It can potentiate non-canonical Wnt pathways, such as the Wnt/Planar Cell Polarity (PCP) and Wnt/Ca2+ pathways (zhou et al., 2017) Zhou et al., 2017). Furthermore, in some cellular environments, DKK3 may even enhance canonical signaling, possibly by interacting with other cell surface components like heparan sulfate proteoglycans (HSPGs) to modulate ligand-receptor interactions or by disrupting negative feedback loops. This dual capacity for inhibition and activation makes DKK3 a unique rheostat for Wnt signaling, with its ultimate effect critically dependent on the cellular repertoire of receptors, co-factors, and the specific Wnt ligands present.

A seminal discovery that reshaped the understanding of DKK3 biology was its identification, alongside DKK1, as a functional ligand for the multifunctional receptor LRP1 (Yang et al., 2025). LRP1 is a large endocytic receptor with over 500 known ligands, playing pivotal roles in lipid metabolism, clearance of protein aggregates (like amyloid-β, Aβ), protease regulation, and intracellular signal transduction. DKK3 binds directly to the ligand-binding clusters of LRP1. This interaction is biologically significant as it creates a competitive landscape. For example, in Alzheimer’s disease models, DKK3 competes with Aβ for binding to LRP1, thereby inhibiting the critical LRP1-mediated clearance of Aβ across the blood-brain barrier and contributing to pathological accumulation (Yang et al., 2025). This establishes the DKK3-LRP1 axis as a major regulatory node in proteostasis and neurodegeneration. The relevance to cardiovascular disease is profound. LRP1 is abundantly expressed on vascular smooth muscle cells, endothelial cells, macrophages, and hepatocytes. In VSMCs, LRP1 is essential for maintaining vascular integrity and suppressing abnormal proliferation. Its endocytic function regulates platelet-derived growth factor (PDGF) receptor signaling. The DKK3-LRP1 interaction could therefore directly influence VSMC phenotype, migration, and vascular remodeling, as suggested by its role in AAA (Cao et al., 2025). In endothelial cells, LRP1 regulates angiogenesis, permeability, and inflammatory responses. Furthermore, in the liver, LRP1 is a key receptor for chylomicron remnant clearance (Guo et al., 2024). The ability of DKK3 to modulate LRP1 function implicates it in broader processes of lipid metabolism, atherosclerosis, and vascular inflammation. The compound SJ-300, identified as a selective inhibitor of the DKK3-LRP1 interaction, provides a pharmacological tool to probe this axis and has shown promise in pre-clinical models of Alzheimer’s, highlighting its therapeutic potential (Yang et al., 2025).

Beyond Wnt and LRP1, DKK3 engages in extensive crosstalk with the transforming growth factor-beta (TGF-β) pathway. This interaction appears central to DKK3’s pro-fibrotic actions across multiple organ systems. The relationship is complex and bidirectional. In models of radiation-induced skin fibrosis, DKK3 expression in keratinocytes was shown to drive a TGF-β-mediated activation of Wnt signaling, leading to macrophage polarization towards a pro-fibrotic M2 phenotype and subsequent dermal fibrosis (Li et al., 2026). Conversely, in vascular smooth muscle cells within the context of abdominal aortic aneurysm, DKK3 deficiency led to the activation of a specific branch of the TGF-β pathway (TGFβ3-Smad2/3), which helped maintain a contractile, non-destructive VSMC phenotype (Cao et al., 2025). This suggests that DKK3 can either facilitate or suppress TGF-β signaling outputs in a cell type and context-specific manner, often by modulating the activity of key upstream regulators like ATF6, an ER stress sensor.

### 2.2 DKK3 as a stress biomarker: lessons from the kidney

The most advanced clinical application of DKK3 is as a urinary biomarker (Sun et al., 2025). Elevated uDKK3 strongly predicts future glomerular filtration rate (GFR) loss in diverse populations, including the general population (Norvik et al., 2025), patients with type 2 diabetes (Keller et al., 2026), and children with CKD (Boeckhaus et al., 2026). In the 3,232-patient primary care diabetes cohort, uDKK3 >200 pg/mg Cr added predictive value for a composite renal endpoint on top of albuminuria and estimated glomerular filtration rate (eGFR) (Keller et al., 2026). The significance for cardiology is profound. CKD is a major risk multiplier for CVD (Sun et al., 2025). uDKK3 identifies diabetic patients at high risk for both renal and cardiovascular events (Keller et al., 2026). This suggests DKK3 marks a “high-risk phenotype” of generalized microvascular and parenchymal stress relevant to cardiorenal syndrome. Furthermore, in heart failure (HF) patients, urinary biomarkers like DKK3 are part of novel cardiorenal risk scores (Hahnel et al., 2025). Treatment with the SGLT2 inhibitor empagliflozin increased uDKK3 by 22% in the EMPA-KIDNEY trial, while reducing uromodulin and albuminuria (Malijan et al., 2025). This dissociation (increased tubular stress marker with improved long-term outcomes) underscores the complexity of interpreting biomarker changes and may reflect an initial hemodynamic or adaptive tubular response to therapy.

### 2.3 Pro-fibrotic roles in non-cardiovascular systems

DKK3 drives fibrosis in multiple organs, offering a mechanistic blueprint for its potential cardiac actions. In radiation-induced skin injury, basal keratinocyte-derived DKK3 is a pivotal regulator. Its increase leads to ROS, TGF-β/Wnt activation, epidermal hyperplasia, macrophage polarization towards a pro-fibrotic (high CD163 and CD206) M2 phenotype, myofibroblast activation, and dermal fibrosis. Genetic deletion of DKK3 in keratinocytes markedly reduced these effects (Li et al., 2026). In pancreatic cancer, it preserves acinar integrity early *via* MEK-Fos signaling but later, when expressed by cancer-associated fibroblasts (CAFs), promotes tumor aggression and fibrosis *via* IL-6-JAK-STAT3 signaling (Srinivasan et al., 2026). Hepatic stellate cell activation and liver fibrosis are also associated with altered DKK3 expression (Liu et al., 2022). Across these systems, DKK3 is induced by injury/stress in parenchymal cells, modulates Wnt/TGF-β crosstalk, recruits and reprofiles immune cells (especially macrophages), and activates fibroblasts. These are hallmark processes in cardiac remodeling (myocardial fibrosis, adverse stiffening) and vascular remodeling (neointima formation, aneurysm wall degeneration).

### 2.4 DKK3 in inflammation and immune modulation

Beyond its direct effects on parenchymal cells, DKK3 functions as a potent immunomodulatory cytokine that bridges cellular stress to innate and adaptive immune responses—a role that is indispensable for understanding its pro-fibrotic and context-dependent actions across organ systems.​

In a radiation-induced skin injury model, keratinocyte-derived DKK3 promoted a TGF-β/Wnt signaling cascade that drove macrophage polarization toward a pro-fibrotic M2 phenotype, characterized by elevated CD163 and CD206 surface expression, concomitant with myofibroblast activation and dermal fibrosis (Li et al., 2026); conversely, DKK3 genetic deletion shifted macrophages toward an M1-like phenotype and attenuated fibrosis. In the context of pancreatic cancer, DKK3 expression in cancer-associated fibroblasts (CAFs) promoted tumor progression and fibrosis through activation of the IL-6-JAK-STAT3 signaling axis, with downstream pro-migratory and pro-fibrotic gene programs reversible by pharmacological STAT3 inhibition. These findings establish DKK3 as a paracrine orchestrator of the macrophage–fibroblast crosstalk that defines chronic inflammatory fibrosis (Li et al., 2026). DKK3 also directly regulates T-cell responses in a context-dependent manner. In experimental autoimmune encephalomyelitis (EAE), DKK3 was upregulated in the CNS in an IFN-γ-dependent manner and functioned as a negative feedback regulator that suppressed local CD4^+^ and CD8^+^ T-cell reactivity; genetic deletion or antibody-mediated neutralization of DKK3 exacerbated EAE with increased IFN-γ-producing T-cell infiltration (Meister et al., 2015). Conversely, *in vitro* studies demonstrate that recombinant DKK-3 promotes Th1 polarization of naïve CD4^+^ T cells through upregulation of T-bet and downregulation of GATA-3, while inducing CD40 and CD86 expression on dendritic cells. More recently, blockade of DKK3 in tumor models enhanced CD8^+^ T-cell activation and Th1 differentiation while reducing M2 macrophage and MDSC infiltration, improving response to immune checkpoint blockade (Shi et al., 2025). Thus, DKK3 exhibits dual and opposing immunomodulatory functions: it can act as a tissue-derived suppressor of excessive local T-cell reactivity, yet in other contexts, its inhibition unleashes anti-tumor (and potentially anti-fibrotic) immunity. This duality is central to interpreting DKK3’s net effect in cardiovascular inflammation. In human THP-1 macrophages, DKK3 binds to the CKAP4 receptor and activates the canonical NF-κB pathway *via* an Akt-dependent mechanism, inducing IκBα phosphorylation, p65 nuclear translocation, and upregulation of NF-κB target genes. This DKK3/CKAP4/NF-κB axis drives macrophage activation and downstream CCL4, IL-1β, and CXCL10 expression (An et al., 2026; Wang et al., 2024). Consistent with this, DKK3 deficiency in ApoE^−/−^ mice attenuated atherosclerosis by reducing macrophage inflammatory mediator secretion and inactivating NF-κB signaling within plaques (Cheng et al., 2017; Karamariti et al., 2018). In pancreatic CAFs, DKK3 drives IL-6-JAK-STAT3 signaling to promote fibrosis and tumor aggressiveness (Srinivasan et al., 2026). Collectively, these data position DKK3 as a stress-induced rheostat that couples parenchymal injury to NF-κB- and JAK-STAT3-driven inflammatory and fibrotic programs, with the net immunological output dictated by the cellular context, disease stage, and DKK3 concentration.

The anti-aging protein Klotho provides a conceptual mirror to DKK3 in inflammation and fibrosis. Klotho exerts anti-inflammatory and anti-fibrotic effects by inhibiting TGF-β, Toll-like receptor (TLR), and NF-κB signaling pathways, while simultaneously modulating the phenotype and function of monocytes, macrophages, T cells, and B cells. In stark contrast, DKK3 often acts in opposition—promoting NF-κB activation, driving M2 macrophage polarization, and activating IL-6-JAK-STAT3 signaling to amplify fibrosis (Zhao et al., 2024). This functional antagonism offers a conceptual framework for understanding DKK3’s context-dependent duality: in conditions where Klotho is downregulated (e.g., chronic kidney disease, metabolic syndrome), unrestrained DKK3 signaling may tip the immune milieu toward chronic inflammation and fibrosis. Conversely, in acute injury settings (e.g., ischemic stroke, post-MI myocardium), DKK3’s protective, anti-apoptotic actions may dominate, illustrating that the DKK3–Klotho axis embodies a Yin-Yang balance in stress response biology. This immunological dichotomy—between DKK3 as a homeostatic brake and as a driver of chronic inflammation—must be carefully dissected when considering DKK3 as a therapeutic target in cardiovascular disease.

## 3 DKK3 in cardiovascular diseases: evidence as a biomarker

### 3.1 The renal-cardiac axis: uDKK3 for cardiovascular risk stratification

As detailed in Section 2.2, uDKK3 is a robust biomarker of renal tubular stress and a predictor of CKD progression. Given the powerful bidirectional link between kidney and heart function (cardiorenal syndrome), biomarkers of renal pathology inherently inform cardiovascular risk (Raveendra et al., 2026). In the diabetic cohort study by Keller et al., elevated uDKK3 was associated not only with renal progression but also with an increased risk for adverse cardiovascular events independently of traditional risk factors (Keller et al., 2026). This positions uDKK3 as a marker of systemic microvascular and endothelial dysfunction, a common substrate for both diabetic nephropathy and cardiovascular disease. Patients with acute decompensated heart failure (ADHF) have a high incidence of concomitant renal dysfunction. A pilot study investigating biomarkers in ADHF included DKK3 among a panel of novel markers. While the primary risk scores (ELAN-HF, ADHF/NT-proBNP) showed fair predictive value for 90-day mortality, the study suggested that a multi-marker panel including cardiorenal biomarkers could improve risk prediction (Hahnel et al., 2025). Larger studies are needed to define the specific additive value of circulating (plasma/serum) DKK3 over established cardiac biomarkers like NT-proBNP in heart failure populations.

### 3.2 Circulating DKK3 in primary cardiovascular conditions and future directions

Research on plasma or serum DKK3 levels in primary CVDs—such as coronary artery disease (CAD) and HF—is indeed less developed compared to the well-established role of uDKK3 in nephrology. As noted in the document, the reviewed materials do not contain large-scale clinical studies directly linking plasma DKK3 with incident myocardial infarction or HF, highlighting a significant knowledge gap. However, emerging clinical evidence beyond the provided documents, coupled with mechanistic insights, provides a compelling rationale for DKK3 as a circulating biomarker in cardiology.

#### 3.2.1 Emerging clinical evidence beyond the provided documents

To address the identified gap, recent studies have begun to directly investigate circulating DKK3 in cardiovascular contexts, yielding nuanced and sometimes contradictory results that highlight the complexity of its role. Supporting a mechanistic link to vascular inflammation, a study on periodontitis and CAD found elevated DKK3 protein levels and gene expression in patients with both conditions, which decreased after periodontal therapy, suggesting DKK3 may reflect inflammatory burden in the vasculature. However, the relationship between systemic DKK3 levels and atherosclerosis appears complex. In an asymptomatic Chinese cohort (Wang et al., 2020), lower serum DKK3 levels were independently associated with a higher risk of significant coronary stenosis (≥50% luminal narrowing). Participants in the lowest DKK3 tertile had a 2.42-fold increased risk of significant stenosis compared to those in the highest tertile (OR: 2.42; 95% CI: 1.10–5.33). This finding challenges a simple linear correlation and suggests DKK3 may have context-dependent roles, potentially serving as a protective factor in early subclinical atherosclerosis in certain populations (Mahendra et al., 2025). Investigations into DKK3 in HF present a mixed picture regarding its independent prognostic value. While one study confirmed that plasma DKK3 concentrations are higher in HF patients compared to controls (46.4 ng/mL vs. 36.3 ng/mL; *P* < 0.001), this elevation was strongly associated with HF risk factors and comorbidities such as age, kidney function, and atrial fibrillation. Crucially, after correction for established prediction models, DKK3 did not independently predict the composite outcome of all-cause mortality or HF hospitalization (HR 1.13 per DKK3 doubling; 95% CI: 0.79–1.61; *P* = 0.503) (Piek et al., 2020). This indicates that while DKK3 is elevated in HF, its prognostic value may not be incremental to established clinical factors and biomarkers like natriuretic peptides. Conversely, urinary DKK3 (uDKK3) shows promise as a prognostic marker specifically for renal function decline in HF patients. Higher baseline uDKK3 levels are associated with a greater subsequent decline in eGFR, even in patients without chronic kidney disease (Pieper et al., 2024). However, the interpretation of this negative result must be tempered by the findings of more recent large-scale studies. In the SPRINT trial, elevated urinary DKK3 (uDKK3) was independently associated with a higher risk of cardiovascular events, end-stage kidney disease, and all-cause mortality in hypertensive adults, even after adjustment for eGFR and albuminuria (Peschard et al., 2024). Furthermore, Piek et al. themselves subsequently reported in a general population cohort that higher plasma DKK3 levels were significantly associated with incident cardiovascular disease, suggesting that the lack of independent prediction in a prevalent HF cohort may reflect disease-specific confounding rather than a universal null association (Piek et al., 2021). These data highlight the need for careful contextualization of biomarker performance across different populations and specimen types.

#### 3.2.2 Integration with document-based insights and future biomarker research needs

The findings above begin to fill the documented knowledge gap. When combined with the provided document’s insights—such as the elevation of DKK3 in modulated VSMCs in human AAA tissue (Cao et al., 2025) and its link to periodontitis-associated CAD (Mahendra et al., 2025)—a coherent picture emerges. Circulating DKK3 appears to be a marker of active vascular smooth muscle cell phenotypic modulation, vascular inflammation, and adverse myocardial remodeling. To definitively establish the clinical utility of circulating DKK3, the following targeted future research is urgently needed. Conduct well-powered, multicenter prospective cohort studies to validate if plasma/serum DKK3​ independently predicts: Incident HF:​ In population-based cohorts, especially in pre-HF stages (Stage A/B); HF Progression:​ In existing HF cohorts, with explicit subgroup analyses by HF subtype (HFrEF vs. HFpEF) and kidney function strata, differentiating its value in HFrEF vs. HFpEF. Given DKK3’s pro-fibrotic role, it may be particularly relevant in HFpEF. Studies should report adjusted HRs with confidence intervals for clinically meaningful increments (e.g., per doubling of concentration or by quartile); Atrial Fibrillation (AF) Incidence: Given the role of atrial fibrosis; Post-AMI Remodeling:​ As a specific marker for predicting left ventricular systolic dysfunction or adverse remodeling (e.g., >15% increase in LV end-systolic volume) 6–12 months post-MI.

## 4 DKK3 in cardiovascular pathophysiology: insights from basic and translational research

### 4.1 Vascular diseases

The most direct and compelling evidence for a pathogenic role of DKK3 in CVD comes from research on AAA. Single-cell RNA sequencing of human AAA tissue revealed that DKK3 is significantly upregulated specifically in phenotypically switched VSMCs, highlighting its association with a key cellular driver of the disease (Cao et al., 2025). This finding is robustly supported by genetic interrogation in preclinical models. Using the standard angiotensin II-infused Apoe−/− mouse model of AAA, systemic knockout of Dkk3 markedly decreased both AAA incidence and rupture-related mortality. Importantly, VSMC-specific knockdown of Dkk3 recapitulated this protective effect, and reintroduction of DKK3 *via* viral vectors exacerbated AAA development, confirming DKK3 as an active promoter of the disease within the vascular wall. Mechanistically, DKK3 drives the deleterious phenotypic switch of VSMCs from a contractile, quiescent state to a synthetic, proliferative, and inflammatory phenotype, characterized by increased production of matrix-degrading enzymes like matrix metalloproteinases (MMPs). At the molecular level, DKK3 promotes this synthetic phenotype by suppressing a novel ATF6-TGFβ3-Smad2/3 signaling axis; DKK3 deficiency leads to downregulation of the ER stress sensor ATF6, which in turn results in activation of TGFβ3-Smad2/3 signaling that helps maintain the contractile VSMC phenotype and inhibits destructive MMP production (Cao et al., 2025). Collectively, these data unequivocally identify DKK3 as a critical promoter of AAA pathogenesis and a compelling therapeutic target for stabilizing aneurysms. In stark contrast to its uniformly pathogenic role in AAA, DKK3 exhibits a complex and context-dependent function in atherosclerosis, acting as both a protective factor and a modulator of plaque composition. Epidemiological data from the prospective Bruneck Study demonstrated that higher plasma DKK3 levels are independently associated with reduced carotid artery intima-media thickness and slower progression of atherosclerosis, positioning DKK3 as an atheroprotective factor (Yu et al., 2017). Experimentally, this is supported by findings that Dkk3 deficiency in Apoe−/− mice accelerates atherosclerotic lesion development and, following vascular injury, impairs re-endothelialization while exacerbating neointima formation (Yu et al., 2017). The protective mechanism involves DKK3 enhancing endothelial cell migration and repair *via* activation of the ROR2/Dvl1/Rac1/JNK signaling pathway (Yu et al., 2017). However, DKK3’s role extends beyond endothelial repair to directly influence plaque stability. While its absence leads to vulnerable plaques with reduced smooth muscle cell (SMC) content and increased hemorrhage (Karamariti et al., 2018), its presence can induce the differentiation of vascular progenitors and fibroblasts into SMCs *via* TGF-β/ATF6 and Wnt pathways, thereby increasing plaque SMC numbers and extracellular matrix, which may stabilize lesions (Karamariti et al., 2018). This dual capacity—promoting endothelial healing and modulating SMC differentiation—underscores DKK3 as a multifaceted regulator of atherosclerotic plaque phenotype, with its net effect likely dependent on the disease stage and cellular microenvironment. Beyond atherosclerosis, a growing body of evidence directly demonstrates that DKK3 plays a crucial and active role in promoting endothelial function, regeneration, and angiogenesis, contradicting the notion that evidence is merely indirect. DKK3 enhances endothelial cell migration, a key process in vascular repair, as outlined above (Yu et al., 2017). Furthermore, it exerts potent pro-angiogenic effects. In cultured human endothelial cells, DKK3 upregulates vascular endothelial growth factor (VEGF) expression and secretion by activating the activin receptor-like kinase 1 (ALK1)/Smad1/5/8 pathway, directly stimulating tube formation (Busceti et al., 2017). Its pro-angiogenic function is also regulated post-transcriptionally, as it is a direct target of the microRNA miR-92a-3p contained in tumor-derived extracellular vesicles; suppression of DKK3 by miR-92a-3p facilitates angiogenesis (Yamada, Heishima, Akao and Senda, 2019). Remarkably, DKK3 possesses the capability to directly reprogram somatic cells into endothelial lineage. Under defined conditions, DKK3 overexpression can transdifferentiate human fibroblasts into functional endothelial progenitor cells and subsequently into mature endothelial cells capable of forming patent monolayers, a process regulated by the VEGF/miR-125a-5p/Stat3 axis (Chen et al., 2019). This highlights a previously underappreciated role for DKK3 in endothelial regeneration and tissue engineering. The functional significance of DKK3 in endothelial homeostasis is further underscored by findings in the renal vasculature, where exosomal DKK3 derived from stressed tubular epithelial cells can influence glomerular endothelial cell fate (Cao et al., 2025). Collectively, these studies reveal DKK3 as a multifunctional cytokine that enhances endothelial migration, drives angiogenesis through VEGF induction, and even directs cellular reprogramming to an endothelial fate, presenting significant therapeutic potential for diseases characterized by endothelial dysfunction or loss (Figure 2).

**FIGURE 2:**
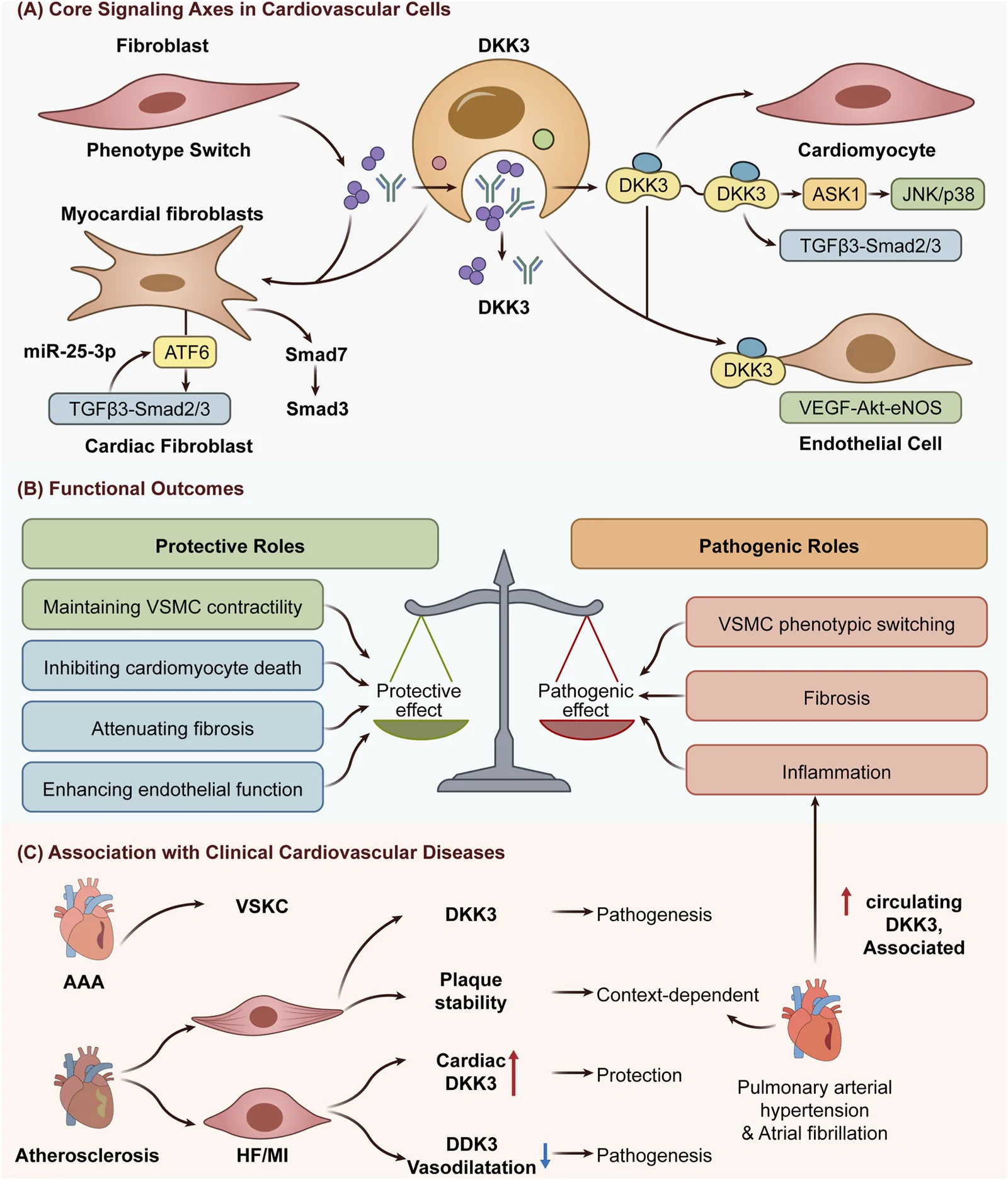
The context-dependent roles and molecular mechanisms of DKK3 in cardiovascular pathophysiology. This schematic summarizes the dual and cell-type-specific functions of Dickkopf-3 (DKK3) in the cardiovascular system, as detailed in Section 4. **(A)** Core Signaling Axes in Cardiovascular Cells: Secreted DKK3 acts on major cardiac and vascular cell types through distinct pathways. In vascular smooth muscle cells (VSMCs), DKK3 suppresses the ATF6-TGFβ3-Smad2/3 axis, promoting a synthetic, pro-inflammatory phenotype. In cardiomyocytes, DKK3 inhibits the ASK1-JNK/p38 cascade, protecting against apoptosis and adverse remodeling. In cardiac fibroblasts, DKK3 is a target of miR-25–3p; its downregulation leads to reduced Smad7, enhancing pro-fibrotic Smad3 signaling. In endothelial cells, DKK3 activates the VEGF/Akt/eNOS pathway, promoting vasodilation and repair **(B)** Functional Outcomes: A Delicate Balance: The activation of these pathways leads to divergent functional outcomes. DKK3 can exert protective roles (left panel), such as maintaining VSMC contractility, inhibiting cardiomyocyte death, attenuating fibrosis, and enhancing endothelial function. Conversely, in specific disease contexts (e.g., sustained high expression), DKK3 can drive pathogenic processes (right panel), including VSMC phenotypic switching, fibrosis, and inflammation. The net effect, symbolized by the central balance, is highly dependent on the cellular, temporal, and disease context **(C)** Association with Clinical Cardiovascular Diseases: These mechanistic insights correlate with clinical and preclinical observations. DKK3 is a direct pathogenic driver in abdominal aortic aneurysm (AAA). Its role in atherosclerosis is complex, influencing both endothelial protection and plaque phenotype modulation. Within the myocardium, local DKK3 expression is cardioprotective in heart failure and post-infarction models, whereas its circulating form is a biomarker. Deficiency in DKK3 contributes to hypertension. Elevated circulating DKK3 is associated with pulmonary arterial hypertension (PAH) and atrial fibrillation (AF), though its precise pathogenic role in these conditions requires further investigation. This figure underscores the principle of context-duality that is central to understanding DKK3 in cardiovascular disease.

### 4.2 Myocardial diseases and heart failure

#### 4.2.1 Divergent evidence: circulating biomarker vs. cardiac protector

The role of DKK3 in myocardial diseases presents a more complex and seemingly contradictory picture compared to its clear pathogenic role in AAA, highlighting the principle of context-duality. Clinical biomarker studies suggest limited utility for circulating DKK3 in HF prognostication. While plasma DKK3 levels are elevated in HF patients compared to controls, this increase is strongly correlated with comorbidities such as age, kidney dysfunction, and atrial fibrillation. After multivariate adjustment, plasma DKK3 did not independently predict the composite outcome of all-cause mortality or HF hospitalization, indicating its prognostic value may not be incremental to established clinical models (Piek et al., 2020). This suggests circulating DKK3 in HF may be a marker of systemic stress and comorbid illness rather than a direct, independent driver of cardiac dysfunction. This large, well-phenotyped cohort study robustly demonstrates the lack of independent prognostic value for plasma DKK3 in HF. A key limitation, however, is the single-center design and the predominantly Caucasian population, warranting external validation. uDKK3 is elevated in HF patients as early as New York Heart Association class I and is a significant predictor of subsequent eGFR decline, a common and prognostically important complication (Pieper et al., 2024). This positions uDKK3 as a valuable biomarker for identifying HF patients at high risk for worsening renal function. In stark contrast to the ambiguous prognostic value of its circulating form, direct experimental evidence from cardiac tissue reveals DKK3 as a potent cardioprotective factor. In a mouse model of myocardial infarction (MI), global Dkk3 deficiency led to increased mortality, larger infarct size, exacerbated left ventricular dysfunction, and heightened inflammation and apoptosis post-MI. Conversely, cardiac-specific overexpression of DKK3 conferred the opposite, protective phenotype (Bao et al., 2015). This cardioprotection is mediated, at least in part, by DKK3’s inhibition of the pro-apoptotic and pro-inflammatory ASK1-JNK/p38 signaling cascade in cardiomyocytes (Bao et al., 2015). This protective role extends to chronic pressure-overload models. In rabbits with volume- and pressure-overload induced chronic HF, treatment with curcumin exerted cardioprotective effects, and these benefits were mechanistically linked to the upregulation of cardiac DKK3 expression, which subsequently inhibited p38 and JNK signaling pathways (Cao et al., 2018). Therefore, while plasma DKK3 may reflect systemic illness, cardiac-derived DKK3 appears to play a direct, beneficial role in mitigating injury, remodeling, and dysfunction following acute and chronic cardiac stress.

#### 4.2.2 Direct antifibrotic mechanisms in the myocardium

Beyond acute injury protection, DKK3 also directly antagonizes the process of myocardial fibrosis, a key pathological substrate in HF. A specific molecular pathway has been elucidated in cardiac fibroblasts. The microRNA miR-25–3p, which is upregulated in the failing myocardium of both mice and humans, directly targets and suppresses Dkk3 expression. The downregulation of DKK3, in turn, leads to reduced expression of Smad7, an inhibitory Smad, resulting in enhanced activation of the pro-fibrotic Smad3 pathway. This sequence ultimately increases the expression of fibrosis-related genes like Col1a1, Col3a1, and Acta2 (encoding α-SMA). Consequently, restoration or enhancement of DKK3 expression can ameliorate Smad3 activation and suppress fibrotic gene expression in cardiac fibroblasts (Zeng et al., 2021). This miR-25-3p-DKK3-Smad7 axis establishes DKK3 as a *bona fide* endogenous brake on the TGF-β/Smad3-driven fibrotic cascade within the heart, providing a precise mechanism for its anti-fibrotic action.

### 4.3 Other related cardiovascular processes

#### 4.3.1 Atrial fibrillation

AF is an advancing fibrosis disease with challenge treatment (Lin et al., 2025). Contrary to the initial assertion that direct evidence is lacking, clinical studies have now established a clear association between circulating DKK3 and AF, positioning it as a potential biomarker in this context. In a prospective cohort of patients with cryptogenic stroke, plasma DKK3 levels were significantly higher in those in whom subsequent intensive monitoring revealed underlying AF. A model combining Angiopoietin-2 and DKK3, along with age and sex, demonstrated good performance for detecting AF in this population [Area Under the Curve (AUC) = 0.733] (Pala et al., 2022). This finding suggests that elevated DKK3 may reflect the atrial stretch, vascular changes, or fibrotic substrate associated with AF pathophysiology. While this clinical correlation is established, the precise cellular role of DKK3 in the atria remains to be fully elucidated. Based on its well-documented pro-fibrotic actions in other organs, it is plausible that DKK3 contributes to the development of the atrial fibrotic substrate that sustains AF. The profibrotic cascade involving TGF-β/Smad signaling and fibroblast activation (Kano et al., 2022), driven by DKK3 in systems like the skin and kidney, is directly translatable to the atrial milieu. It is hypothesized that atrial stretch or metabolic stress could upregulate DKK3 in atrial fibroblasts or cardiomyocytes, promoting fibrosis in a paracrine manner. Future research should build on the biomarker finding by measuring DKK3 expression directly in atrial tissue from patients and employing genetic models to test if Dkk3 deletion attenuates atrial fibrosis and AF inducibility in animals subjected to predisposing stimuli.

#### 4.3.2 Pulmonary arterial hypertension

Emerging clinical evidence also implicates DKK3 in pulmonary arterial hypertension (PAH), extending beyond mechanistic parallels. In patients with precapillary PH, plasma levels of DKK3 are elevated and correlate with disease severity and prognosis. In a cohort study, higher baseline levels of DKK3 were independently associated with increased mortality risk, even after adjustment for hemodynamic parameters and NT-proBNP. Furthermore, an increase in DKK3 levels during follow-up was also independently linked to poor outcomes (Ueland et al., 2021). This positions circulating DKK3 as a prognostic biomarker in PAH. The pathological hallmark of PAH is the phenotypic switching of pulmonary artery smooth muscle cells (PASMCs) to a proliferative state, a process bearing a striking resemblance to the DKK3-driven modulation of vascular smooth muscle cells observed in abdominal aortic aneurysm. The mechanistic finding that DKK3 promotes a synthetic VSMC phenotype by suppressing the ATF6-TGFβ3-Smad2/3 axis is therefore highly relevant to PAH (Cao et al., 2025). One can hypothesize that in PAH, stressors like hypoxia and inflammation upregulate DKK3 in PASMCs, which then inhibits protective, contractile-maintaining pathways, thereby unleashing pathological proliferation and migration. This is supported by basic research showing that extracellular DKK3 can interact with proteins like transforming growth factor beta induced protein (TGFBI) to modulate cellular adhesion and motility through Focal Adhesion Kinase (FAK) signaling, processes critical for vascular cell behavior (Kano et al., 2022). Therefore, investigating DKK3 expression in PAH models and testing the efficacy of DKK3-modulating strategies on pulmonary vascular remodeling are clear future directions warranted by both clinical biomarker data and strong mechanistic rationale.

### 4.4 Metabolic cardiovascular disease: DKK3 as a link between dysmetabolism and vascular injury

DKK3 is deeply implicated in the pathogenesis of metabolic cardiovascular diseases, yet its role transcends that of a simple pathogenic driver. Emerging evidence delineates a dual identity: DKK3 serves as a sensitive biomarker of metabolic and cellular stress, while simultaneously functioning as a critical protective factor for cardiovascular homeostasis. Its actions are highly context-dependent, influenced by the metabolic milieu, target tissue, and disease stage, making it a complex but compelling node in the cardiometabolic axis (Table 2).

**TABLE 2:** Critical synthesis: Context-dependent roles of DKK3 in major cardiovascular conditions.

Disease context	Predominant role of DKK3	Key supporting evidence	Proposed mechanism	Major unresolved questions/Limitations
Abdominal aortic aneurysm (AAA)	Pathogenic driver	VSMC-specific ↑ in human tissue; genetic knockout prevents AAA in mice	Suppression of ATF6-TGFβ3-Smad2/3, leading to synthetic VSMC phenotype	Translational gap: Efficacy of pharmacological inhibition in advanced human AAA unknown
Atherosclerosis	Context-dual: Protective and modulatory	Plasma level ↓ associates with CAD; knockout accelerates plaques but impairs endothelial repair	Endothelial protection *via* VEGF/Akt/eNOS vs. plaque modulation *via* SMC differentiation	Net effect on clinical atherothrombosis (plaque stability) is unpredictable and stage-dependent
Heart failure (myocardium)	Tissue protector	Cardiac-specific overexpression is protective post-MI; inhibits ASK1-jnk/p38 and Smad3 fibrosis	Cardioprotective anti-apoptotic and anti-fibrotic signaling within cardiomyocytes/fibroblasts	Paradox vs. plasma DKK3: Why is circulating DKK3 elevated but not prognostic? Source and sink relationship unclear
Hypertension	Protective factor	Genetic deletion causes hypertension; central/peripheral DKK3 enhances NO bioavailability	Activation of VEGF/Akt/eNOS axis in endothelium and CNS.	Therapeutic potential of DKK3 augmentation in human hypertension remains unexplored
Diabetic cardiorenal Disease​	Biomarker (passenger?)	uDKK3 strongly predicts renal/CVD events; ↑ with empagliflozin (beneficial therapy)	Marker of tubular stress; role in cardiac fibrosis may be protective	EMPA-KIDNEY paradox: Is DKK3 a causal driver or a stress-response bystander in CKD progression?

#### 4.4.1 DKK3 in diabetic vasculopathy, cardiac remodeling, and fibrosis

The role of DKK3 in the fibrotic complications of diabetes is characterized by significant context duality, differing markedly between the renal and cardiac compartments. Elevated uDKK3 robustly predicts the progression of diabetic kidney disease and, importantly, also identifies patients with type 2 diabetes at higher risk for adverse cardiovascular events, underscoring its value in marking a high-risk cardiorenal phenotype (Keller et al., 2026). In striking contrast, within the heart under metabolic or hemodynamic stress, DKK3 often acts as a protective brake against maladaptive remodeling. In a model of angiotensin II (Ang II)-induced cardiac injury, overexpression of DKK3 significantly attenuated cardiac hypertrophy and interstitial fibrosis. This cardioprotection was mediated through two interconnected pathways: (1) inhibition of the disintegrin and metalloproteinase ADAM17, leading to increased levels of the protective enzyme angiotensin-converting enzyme 2 (ACE2), and 2) suppression of the pro-fibrotic GSK-3β/β-catenin signaling pathway (Zhai et al., 2018). This identifies cardiac DKK3 as a beneficial modulator of the renin-angiotensin system and Wnt signaling. Similarly, DKK3 has been shown to protect against post-myocardial infarction remodeling by inhibiting pro-apoptotic kinase pathways. However, this protective role can be compromised. For instance, exposure to environmental nano-microplastics (NMPs) induces myocardial fibrosis, in part, by downregulating cardiac DKK3 expression. This DKK3 deficiency leads to impaired mitophagy and subsequent activation of the NLRP3 inflammasome, promoting cardiomyocyte pyroptosis and fibrosis (Xiong, Xiong, Hua, Chen and Wang, 2025). This indicates that the loss of cardioprotective DKK3, rather than its overabundance, can be a driver of injury in certain metabolic-toxic stress contexts.

#### 4.4.2 DKK3 as a metabolic myokine in obesity and insulin resistance

Beyond the classic cardiovascular organs, DKK3 is now recognized as a “myokine”—a cytokine secreted by skeletal muscle—with important implications for systemic metabolism. In obesity and type 2 diabetes, DKK3 expression in skeletal muscle is significantly upregulated, and its circulating levels are elevated (Z. T. Chen, Weng, Lin and Meng, 2024; Xu et al., 2023). Functionally, muscle-derived DKK3 acts in a paracrine manner to inhibit the differentiation of muscle stem cells (satellite cells), thereby impairing muscle regeneration and contributing to the decline in muscle fitness associated with metabolic disease (Xu et al., 2023). This establishes DKK3 as a mediator of the detrimental crosstalk between dysfunctional muscle and impaired tissue repair in the metabolic state. The regulation of muscle DKK3 involves the transcriptional coactivator Baf60c, which interacts with Six4 to suppress Dkk3 expression; this repression is lost in obesity, leading to elevated DKK3 (Xu et al., 2023).

## 5 DKK3 as a therapeutic target: potential and challenges in cardiovascular medicine

The dual identity of DKK3 as both a biomarker of organ stress and a direct pathogenic mediator naturally propels it into the realm of therapeutic investigation. Modulating the DKK3 axis presents a tantalizing opportunity to intervene in the core processes of fibrosis, vascular remodeling, and metabolic dysfunction that underlie many CVDs. However, the path from mechanistic insight to viable therapy is fraught with complexities intrinsic to DKK3’s biology. This section expands on the potential therapeutic strategies, provides a critical analysis of the formidable challenges supported by concrete data, and explores the innovative concept of companion diagnostics.

### 5.1 Therapeutic strategies: from preclinical tools to clinical trials

#### 5.1.1 Inhibition strategies

For conditions where DKK3 acts as a pathogenic driver (e.g., late-stage fibrosis, AAA), suppression of its activity is the primary goal. The development of high-affinity monoclonal antibodies (mAbs) against DKK3 is a logical step. Preclinical proof-of-concept is strong. In the AAA model using Angiotensin II-infused Apoe−/−mice, systemic genetic deletion of Dkk3 reduced AAA incidence from approximately 80% in wild-type controls to about 30% (Dkk3−/−Apoe−/−), and more importantly, completely eliminated rupture-related mortality over the study period (0% vs. ∼40% in controls) (Cao et al., 2025). This dramatic effect provides a clear therapeutic benchmark for a neutralizing antibody. The predicted efficacy of a therapeutic mAb would aim to recapitulate this protection, potentially reducing AAA expansion rate (e.g., by >50% as measured by serial ultrasound) in preclinical models. Targeting the specific interaction between DKK3 and its receptor LRP1 represents a more refined approach. The compound SJ-300 was identified as a selective inhibitor of the DKK3-LRP1 interaction. In an Alzheimer’s disease mouse model (APP/PS1), chronic administration of SJ-300 (10 mg/kg, i. p., five times per week for 3 months) successfully restored amyloid-β (Aβ) clearance across the blood-brain barrier. This was evidenced by a significant ∼40% reduction in insoluble Aβ42 levels in the brain cortex and a concomitant improvement in cognitive function in the Morris water maze test (Yang et al., 2025). This study is critical as it provides direct pharmacological evidence that disrupting the DKK3-LRP1 axis is feasible and therapeutically beneficial in a chronic disease model, establishing a precedent for its use in vascular pathologies where this axis is relevant. Transient knockdown of DKK3 using small interfering RNA (siRNA) or antisense oligonucleotides (ASOs) offers a strategy with potential for organ-selective delivery. In the same AAA study, VSMC-specific Dkk3knockdown using Cre-Lox technology phenocopied the protective effect of global knockout, confirming the vascular wall as a key site of action (Cao et al., 2025). The development of siRNA conjugated to ligands targeting vascular smooth muscle cells or formulated in nanoparticles for localized perivascular delivery could minimize off-target effects.

#### 5.1.2 Augmentation/replacement strategies

In contexts where DKK3 loss or deficiency is pathogenic, restoration of its function is the goal. This is best exemplified in oncology, where DKK3 acts as a tumor suppressor. The most advanced clinical application of a DKK3-based therapy is in cancer. A Phase I/II clinical trial investigated intrapleural administration of an adenoviral vector expressing DKK3 (Ad-SGE-REIC/Dkk3) in combination with the immune checkpoint inhibitor nivolumab for malignant pleural mesothelioma. The therapy demonstrated a manageable safety profile. Among 27 evaluable patients, the disease control rate was 77.8%, with two partial responses and 19 cases of stable disease. The median overall survival was 18.2 months, which compared favorably to historical controls (Jang et al., 2025). This trial is seminal as it provides clinical-grade evidence for the safety and potential efficacy of localized DKK3 gene augmentation, offering a delivery and safety blueprint that could be adapted for cardiovascular applications (e.g., localized delivery to the vascular wall or epicardium) if a pro-DKK3 strategy were ever deemed beneficial. The phase I/II trial provides crucial proof-of-concept for the safety of localized DKK3 gene delivery. However, the open-label design and lack of a placebo-controlled arm limit definitive efficacy conclusions, highlighting the need for larger randomized trials.

### 5.2 Application in specific cardiovascular diseases: a data-driven outlook

The preclinical data is unequivocal and sets a high bar for therapeutic development. A DKK3-neutralizing antibody should be tested in the Ang-II Apoe−/−model with primary endpoints being aortic diameter expansion (target: >70% reduction vs. IgG control) and rupture-free survival (Cao et al., 2025). The mechanism is clear: inhibiting DKK3 would be predicted to de-repress the ATF6-TGFβ3-Smad2/3 pathway in VSMCs, increasing contractile gene expression (e.g., increasing α-SMA and SM22α mRNA by 2–3 fold) and decreasing destructive MMP activity (e.g., reducing MMP2/9 activity by >50%). The rationale is strong but complicated by temporal context duality. The pancreatic cancer study (Srinivasan et al., 2026) is a crucial cautionary tale: early, transient DKK3 expression in acinar cells was protective against inflammation, while its sustained expression in cancer-associated fibroblasts (CAFs) later drove aggression and fibrosis *via* IL-6-JAK-STAT3. In a model of pressure-overload cardiac hypertrophy, analogous temporal profiling is needed. A therapeutic anti-DKK3 antibody might need to be administered only after the transition from adaptive hypertrophy to maladaptive fibrosis, which is a significant clinical challenge. In a murine unilateral ureteral obstruction (UUO) model of renal fibrosis, Dkk3knockout reduced renal collagen deposition by approximately 60% (Srinivasan et al., 2026), supporting the anti-fibrotic potential of inhibition in a pure fibrosis setting. The effect is uncertain and must be empirically determined. VSMC phenotypic modulation is a double-edged sword in plaques. Synthetic VSMCs contribute to plaque growth, but they also form the protective fibrous cap. A study in an atherosclerosis model (e.g., Ldlr−/−mice on high-fat diet) treating with a DKK3 inhibitor would need to carefully assess not just plaque area (which could decrease), but more importantly, plaque stability metrics: fibrous cap thickness (which must not decrease), necrotic core size, and macrophage content. The net effect on clinical endpoints like atherothrombosis is unpredictable.

### 5.3 Major challenges and considerations: beyond mechanism

#### 5.3.1 The paramount challenge: context-duality

This is the single greatest hurdle for DKK3 therapeutics, underscored by starkly opposing data across disease models. For pathogenic role, in AAA (Cao et al., 2025), late-stage pancreatic cancer (Srinivasan et al., 2026), and skin fibrosis (Li et al., 2026), DKK3 promotes disease. For protective role, in ischemic stroke, DKK3 was downregulated and its overexpression was protective, reducing infarct volume by ∼30% in a mouse model (Zhou et al., 2025). In early pancreatic injury, DKK3 preserved acinar integrity. In cancer immunotherapy, anti-DKK3 blockade enhanced the efficacy of anti-PD-1 therapy, increasing tumor-infiltrating CD8^+^ T cells (Shi et al., 2025). Therefore, a “one-size-fits-all” DKK3 modulator does not exist. Therapeutic strategies must be exquisitely disease- and stage-specific. This necessitates a deep understanding of the temporal dynamics of DKK3 expression and function in each cardiovascular condition, which is currently lacking for most.

Translating this challenge into actionable strategy requires a spatiotemporal intervention framework. The 2025 single-cell RNA-seq study by Cao et al. (Cao et al., 2025) provides the first direct evidence for such an approach in AAA: DKK3 expression in the murine AAA model exhibits distinct temporal dynamics, with differential DKK3 upregulation in aortic cells at 7, 14, and 28 days post Ang-II infusion, and single-cell profiling identifying the modulated VSMC cluster as the dominant cellular source of DKK3 throughout disease progression. This temporal and cellular resolution offers a template for defining the “therapeutic window.” We propose that future preclinical studies use inducible conditional knockout models—such as *Dkk3* ^*flox/flox*^; *SM22α-Cre* ^*ERT2*^ mice in AAA or *Dkk3* ^*flox/flox*^; *αMHC-Cre* ^*ERT2*^ mice in MI—combined with tamoxifen-staged induction at predetermined post-injury time points (e.g., day 3, 7, 14, or 28). By titrating the timing of DKK3 ablation relative to injury onset, one can empirically map the boundary between the “adaptive protection phase” and the “pathogenic driving phase.”

The molecular adjudicator of this boundary appears to be ATF6. Cao et al. demonstrated that DKK3 upregulates ATF6 through dual transcriptional (promoter binding) and post-translational (protein stabilization) mechanisms, and that ATF6 in turn represses TGFβ3 transcription, thereby suppressing Smad2/3 activation and driving VSMC phenotypic switching (Cao et al., 2025). Notably, the ATF6-TGFβ3-Smad2/3 axis exhibits dynamic regulation over the AAA time course. We hypothesize that early after injury, modest DKK3 induction may engage compensatory, protective pathways (e.g., ASK1-JNK/p38 inhibition in cardiomyocytes), whereas sustained or high-level DKK3 expression tips the balance toward pathogenic ATF6 accumulation, synthetic VSMC phenotype switching, and MMP-mediated matrix degradation. The threshold at which ATF6 transitions from a homeostatic ER stress sensor to a pathogenic driver may thus serve as a biomarker-defined entry point for therapeutic DKK3 inhibition. Clinically, this framework implies that DKK3-modulating therapies should not be administered uniformly but rather timed to disease stage. In AAA, for instance, pharmacological DKK3 inhibition (e.g., neutralizing antibody or AAV-shRNA) initiated after the establishment of the synthetic VSMC phenotype (approximately day 14 in the Ang-II model) may recapitulate the protective effect of genetic knockout, whereas early inhibition during the putative adaptive phase could be detrimental. In MI, staged administration guided by serial DKK3 and ATF6 measurements could similarly delineate the window wherein DKK3 augmentation aids repair *versus* the window wherein it promotes maladaptive fibrosis. This spatiotemporal precision—enabled by inducible genetics and informed by single-cell temporal mapping—converts the “context-duality obstacle” from a descriptive caveat into a testable, stage-stratified therapeutic paradigm.

#### 5.3.2 Biomarker vs. target discordance: the empagliflozin paradox

The EMPA-KIDNEY trial revealed a critical and perplexing dissociation: treatment with the renal- and cardio-protective SGLT2 inhibitor empagliflozin increased uDKK3 by 22% despite improving long-term renal outcomes (Malijan et al., 2025). If DKK3 were a straightforward pathogenic mediator, inhibiting it should be beneficial, and a therapy that increases it should be harmful. The empagliflozin data contradict this. The rise in uDKK3 may reflect a transient, adaptive tubular stress response to altered hemodynamics and energetics (e.g., tubuloglomerular feedback activation) induced by SGLT2 inhibition, not a pathogenic event. This discordance suggests that for diseases like diabetic kidney disease, inhibiting DKK3 could be ineffective or even detrimental​ if it blunts a potentially adaptive stress-response pathway. It forces a fundamental question: In which diseases is DKK3 a “driver” suitable for inhibition, and in which is it merely a “passenger” biomarker (Table 3)?

**TABLE 3:** Proposed Framework for Discerning the Pathogenic Driver vs. Stress-Response Passenger Roles of DKK3 in Cardiovascular Diseases.

Evaluative criterion	Evidence supporting a ‘driver’ role	Evidence supporting a ‘passenger’ role (biomarker)
Temporal and spatial dynamics	Expression precedes and correlates with the onset/progression of a key pathological process (e.g., fibrosis, inflammation). Cell-type-specific expression localized to the primary disease-driving cells (e.g., VSMCs in AAA, fibroblasts in fibrosis)	Expression changes are a late consequence of established injury or hemodynamic stress. Expression is widespread or primarily in non-parenchymal cells, reflecting a general systemic or organ stress response
Genetic and pharmacological manipulation	Genetic loss-of-function (*in vivo*) attenuates disease development or progression. Pharmacological inhibition (e.g., neutralizing antibody, siRNA) ameliorates pathology in preclinical models	Genetic or pharmacological modulation of DKK3 has minimal or no impact on the core disease phenotype, despite altering its expression levels
Independent prognostic association - plasma DKK3	Circulating DKK3 is an independent predictor of incident CVD in general populations	In prevalent HF, plasma DKK3 loses independent prognostic value after adjusting for NT-proBNP and renal function, suggesting context-dependent utility
Independent prognostic association - urinary DKK3	uDKK3 independently predicts cardiovascular events, ESKD, and mortality in hypertensive and diabetic cohorts	Association may be partially confounded by baseline kidney function; cut-off standardization is lacking
Response to pathogenically-effective therapy	An intervention that improves long-term outcomes leads to a reduction in DKK3 levels, consistent with it being on the causal pathway	An intervention that improves long-term outcomes leads to an increase or no change in DKK3 levels (the “EMPA-KIDNEY paradox”), suggesting its elevation may be part of an acute adaptive stress response dissociated from the chronic disease driver
Mechanistic plausibility and specificity	DKK3 is positioned within a well-defined molecular pathway that is causally linked to the disease pathology (e.g., DKK3-atf6-tgfβ3 axis in VSMC phenotype switch)	The link between DKK3 and disease is correlative, without a direct mechanistic pathway established in the relevant tissue context

The paradox extends to the seemingly contradictory roles of DKK3 in different organ systems. As discussed in Sections 4.2 and 4.4, cardiac-derived DKK3 acts locally to inhibit apoptosis and fibrosis, exerting a protective effect within the myocardium. Conversely, elevated urinary DKK3 (uDKK3), a marker of renal tubular stress, is a strong predictor of both CKD, progression and future cardiovascular events. This apparent contradiction may be explained by compartmentalization. Cardiac DKK3 likely functions in a paracrine/autocrine manner as part of the heart’s local adaptive response to injury. In contrast, elevated systemic (particularly urinary) DKK3 primarily reflects the aggregate burden of cellular stress and injury across multiple organs, most notably the kidneys. This systemic stress burden is itself a powerful driver of adverse cardiovascular outcomes. Thus, DKK3 can simultaneously be a component of the heart’s local defense mechanism and a sensitive biomarker indicating a state of high systemic (e.g., cardiorenal) stress that portends poor prognosis. Disentangling the source (cardiac vs. renal vs. vascular) and the functional consequence (adaptive vs. maladaptive) of circulating DKK3 in a given patient remains a major clinical and research challenge.

#### 5.3.3 Drug development hurdles

From bioavailability and pharmacodynamics, DKK3 is a secreted protein acting both locally and systemically. Effective inhibition may require high systemic drug levels or sophisticated delivery systems to reach target tissues (interstitial space of the heart, vascular wall). The plasma half-life and tissue penetration of biologics (mAbs) or small molecules like SJ-300 need optimization. Global Dkk3knockout mice are viable but exhibit specific phenotypes, most notably asthenozoospermia​ (reduced sperm motility) due to defects in sperm flagellum formation (Xue et al., 2026). While this may reflect a developmental role, it signals that chronic, systemic DKK3 inhibition could have reproductive side effects. Other potential off-target effects in tissues with high basal DKK3 expression (e.g., brain, kidneys) are unknown. Developing tissue-targeted delivery systems (e.g., VSMC- or fibroblast-targeting nanoparticles, AAV vectors with tissue-specific promoters) is likely essential for tolerability.

Targeting DKK3 in cardiovascular disease is a high-risk, high-reward proposition. The mechanistic rationale, particularly in AAA, is exceptionally strong, supported by genetic knockout data showing profound protection. Pharmacological tools (SJ-300) and clinical delivery platforms (Ad-DKK3) exist. However, the path is obstructed by DKK3’s inherent context-dependent duality, starkly illustrated by its protective roles in other diseases and the paradoxical rise in uDKK3 with effective SGLT2 inhibitor therapy. Success will not come from a blunt DKK3 inhibitor but from a precision medicine approach: the concurrent development of a sophisticated companion diagnostic to identify the specific cardiovascular patient population in whom DKK3 is a “druggable” driver of pathology, coupled with targeted delivery systems to maximize efficacy and minimize systemic toxicity. This integrated strategy is essential to translate the compelling biology of DKK3 into a safe and effective cardiovascular therapy.

#### 5.3.4 The uncharted role of sex dimorphism

A critical and largely unaddressed question is whether DKK3 biology exhibits sex-specific differences.​ Cardiovascular diseases, including atherosclerosis, HF, and AAA, as well as fibrotic responses, display well-documented sexual dimorphisms in incidence, progression, and underlying mechanisms, often influenced by sex hormones (Regitz-Zagrosek and Gebhard, 2023). Nowhere is this dimorphism more striking than in AAA, where males exhibit a 4- to 5-fold higher prevalence compared to females, with some studies reporting up to a 10-fold higher risk in men *versus* age-matched women (Pace et al., 2026; Welsh et al., 2025). This epidemiological asymmetry is particularly salient given that DKK3 is a key pathogenic driver of AAA—the marked male predominance raises the provocative hypothesis that androgens may enhance DKK3 expression or signaling output in VSMCs, thereby contributing to the sex bias in disease susceptibility. To evaluate the mechanistic plausibility of this hypothesis, we examined the DKK3 promoter for canonical androgen response elements (AREs). While direct AR-binding peaks at the DKK3 locus have not been prominently reported in ENCODE ChIP-seq datasets from vascular cell types, several lines of evidence support androgen-mediated regulation of DKK3. In prostate epithelial cells, dihydrotestosterone (DHT) induced promoter methylation changes at both the ARSB and DKK3 loci, with DHT exposure suppressing DKK3 expression and thereby sustaining Wnt signaling—an effect reversed by ARSB overexpression, indicating that androgens can transcriptionally modulate DKK3 (Bhattacharyya et al., 2019). Furthermore, DKK3 (as REIC) physically interacts with the small glutamine-rich tetratricopeptide repeat-containing protein α (SGTA), a negative modulator of AR signaling, thereby promoting dynein-dependent AR nuclear transport and upregulating AR signaling output (Kato et al., 2017). We propose that future studies examine DKK3 promoter methylation status and AR occupancy in male vs. female AAA tissues, as well as in VSMCs treated with physiological concentrations of testosterone or DHT, to definitively establish whether the DKK3–androgen axis underlies the sex-specific epidemiology of AAA. We systematically reviewed the published literature on DKK3 in AAA animal models, including the seminal single-cell RNA-seq study by Cao et al. Notably, none of these studies explicitly stratified DKK3 expression or the DKK3–ATF6–TGFβ3–Smad2/3 signaling axis by sex in their animal cohorts. The original reports of Dkk3 deficiency in the Ang-II-infused Apoe^−/−^ AAA model presented pooled data without sex-disaggregated analysis of aneurysm incidence, rupture mortality, or VSMC DKK3 expression levels. This represents a significant methodological limitation, as a true sex-specific effect of DKK3 in AAA cannot be ascertained from the current evidence base. We explicitly call for future preclinical AAA studies to enroll both male and female animals and to report DKK3 expression, signaling activation, and therapeutic responses in a sex-stratified manner. Such analyses are essential to determine whether the profound male predominance of AAA is mechanistically linked to androgen-enhanced DKK3 expression in VSMCs. Currently, there is a paucity of data examining if DKK3 expression, secretion, or signaling function differs between males and females in health or cardiovascular disease states. The existing clinical biomarker studies referenced in this review, such as those in heart failure or atherosclerosis cohorts, have typically either not reported sex-stratified analyses of DKK3 levels or have adjusted for sex as a covariate without exploring potential effect modification. This gap extends to preclinical models, where studies using global or cell-specific Dkk3 knockout mice have generally not reported or investigated potential sex-dependent phenotypes. Therefore, future research must intentionally incorporate sex as a biological variable. This includes conducting sex-stratified analyses in human biomarker studies, employing both male and female animals in preclinical models of cardiovascular disease, and directly testing the interaction between sex hormones and DKK3 regulation in relevant cell types.

### 5.4 Translational perspectives and current limitations

The accumulating evidence positions DKK3, particularly in its urinary form, on the cusp of clinical translation, while highlighting distinct pathways and challenges for its circulating form.

For uDKK3, the most immediate and high-impact application lies in refining risk stratification within the cardiorenal metabolic syndrome, especially in type 2 diabetes. Its ability to identify patients at high risk for both kidney function decline and cardiovascular events, on top of established markers like albuminuria, could guide more personalized surveillance and therapeutic intensification. A practical application could be the use of uDKK3 to enrich clinical trials for novel renal or cardioprotective agents by selecting a population with a high rate of “hard” endpoint progression. Furthermore, serial measurement of uDKK3 might serve as a dynamic biomarker of tubular health in specific settings, such as monitoring for subclinical kidney injury in heart failure management or assessing renal recovery after an acute event. To define the incremental value of uDKK3 over established cardiac biomarkers, future studies must re-evaluate its independent prognostic performance in multivariable models that already include NT-proBNP and high-sensitivity cardiac troponin T (hs-cTnT). Only by demonstrating added discrimination or reclassification beyond these gold-standard markers can uDKK3 claim a unique niche in clinical algorithms. Multi-marker strategies represent a promising direction: Hahnel et al. (Hahnel et al., 2025) explored a panel combining DKK3, sST2, and NGAL for risk stratification in acute decompensated heart failure, and Pala et al. (Pala et al., 2022) successfully used a combination of Angiopoietin-2 and DKK3 to detect occult atrial fibrillation in cryptogenic stroke patients (AUC = 0.733). These examples illustrate that DKK3 may be most useful as part of a composite biomarker signature rather than as a standalone test (Table 4).

**TABLE 4:** Horizontal comparison of cardiorenal stress biomarkers: Induction, pathways, biofluid distribution, and prognostic evidence.

Biomarker	Primary induction conditions	Main signaling pathways	Biofluid(s)	Strength of CV prognostic evidence
DKK3	Tubular/VSMC/cardiomyocyte stress (ER stress, oxidative stress, inflammation)	Wnt modulation, LRP1, ATF6-TGFβ3, ASK1-JNK/p38, IL-6-JAK-STAT3	Urine (validated), plasma/serum (emerging)	Strong for renal/CV events in diabetes (uDKK3); moderate for HF (plasma, non-independent after adjustment); emerging for AAA, AF, PAH
Klotho	Aging, oxidative stress, inflammation, renal injury	Anti-aging *via* FGFR, inhibition of tgfβ/tlr/NF-κb	Plasma/serum, urine	Strong for CKD progression and CV mortality; inverse association with vascular calcification and HF
sST2	Mechanical stretch, inflammation (IL-33 decoy receptor)	IL-33/ST2 signaling (anti-fibrotic when bound, pro-fibrotic when decoy)	Plasma/serum	Strong for HF prognosis (added to NT-proBNP); approved for clinical use in HF
GDF-15	Cellular stress, inflammation, hypoxia, mitochondrial dysfunction	TGFβ superfamily; activates ALK-1/-5, Smad2/3	Plasma/serum	Strong for CV mortality, HF, and bleeding risk; incorporated into some risk scores
Galectin-3	Macrophage activation, fibrosis	Galectin-3 lattice, integrin signaling, TGFβ/Smad	Plasma/serum	Moderate for HF fibrosis and mortality; FDA-cleared but limited incremental value over NT-proBNP

Substantial limitations currently impede routine clinical use. First, there is a lack of standardized, commercially available assays and universally validated diagnostic cut-offs for either urinary or plasma DKK3. Second, the biological heterogeneity of DKK3 is a major interpretative challenge: levels are influenced by age, kidney function, sex, and inflammatory status, requiring careful adjustment in clinical algorithms. Third, the “passenger *versus* driver” dilemma, exemplified by the EMPA-KIDNEY trial where uDKK3 rose with effective therapy, creates uncertainty about whether modulating DKK3 levels is a valid treatment goal or a mere epiphenomenon (Malijan et al., 2025). It is noteworthy that Klotho, another stress-inducible protein with a functional spectrum opposite to that of DKK3, exhibits similar paradoxical behavior: some renoprotective interventions cause bidirectional changes in Klotho levels, suggesting that initial fluctuations of stress-inducible proteins may reflect adaptive tubular epithelial cell responses rather than signals of pathological deterioration (Zhao et al., 2024). Accordingly, the elevation of uDKK3 following SGLT2 inhibitor treatment should not be simplistically interpreted as negative evidence against DKK3 as a therapeutic target; it may instead represent a transient, adaptive stress response dissociated from the chronic disease driver. Finally, demonstrating clinical utility—that measuring DKK3 improves patient outcomes or reduces costs compared to standard care—requires large, prospective, interventional biomarker-guided studies, which have not yet been conducted.

Future translational research must therefore pivot from association to application. Key steps include: 1) Assay standardization across platforms and populations; 2) Prospective validation of proposed uDKK3 cut-offs in diverse, real-world cohorts for hard endpoints; 3) Interventional studies testing if biomarker-guided management (e.g., earlier intervention in high uDKK3 patients) improves outcomes; and 4) Mechanistic studies to decipher the source and functional significance of circulating DKK3 in specific diseases to enable its precise interpretation.

## 6 Summary and conclusion

DKK3 has emerged as a unique, stress-inducible glycoprotein with significant relevance to cardiovascular medicine. It serves as a validated biomarker of renal tubular stress and cardiovascular risk in diabetes, marking a high-risk cardiorenal phenotype. Functionally, DKK3 is an active player in cardiovascular pathophysiology, but its role is highly context-dependent. It is a critical promoter of AAA pathogenesis, a multifaceted regulator of atherosclerosis (both protective and modulatory), a protector of the myocardium against injury and fibrosis, and a guardian of endothelial function and blood pressure. This duality presents both opportunity and challenge for therapy. Success will require a precision medicine approach: developing companion diagnostics to identify patients where DKK3 is a “druggable” driver, coupled with targeted delivery systems to maximize efficacy and minimize toxicity. Future research must prioritize defining the direct, cell-specific roles of DKK3 in the heart and vasculature, clarifying its complex signaling networks, and validating the utility of circulating DKK3 as a clinical biomarker. Filling these gaps will determine if DKK3 can transition from a compelling biological molecule to a bona fide therapeutic target in cardiovascular disease.
